# C-terminus of MUC16 activates Wnt signaling pathway through its interaction with β-catenin to promote tumorigenesis and metastasis

**DOI:** 10.18632/oncotarget.9191

**Published:** 2016-05-05

**Authors:** Qi Liu, Zhen Cheng, Lianzhong Luo, Yun Yang, Zhenzhu Zhang, Huanhuan Ma, Tao Chen, Xi Huang, Shu-Yong Lin, Meijun Jin, Qinxi Li, Xiaotong Li

**Affiliations:** ^1^ State Key Laboratory for Cellular Stress Biology, School of Life Sciences, Xiamen University, Fujian 361102, China; ^2^ Central Laboratory, Xiamen Medical College, Xiamen, Fujian 361008, China; ^3^ School of Basic Medical Sciences, Xinxiang Medical University, Henan 453003, China

**Keywords:** MUC16, C-terminus, β-catenin, Wnt signaling, tumorigenesis

## Abstract

MUC16/CA125 has been identified as a prominent cancer biomarker, especially for epithelial ovarian cancers, in clinical test for over three decades. Due to its huge mass, limited knowledge of MUC16 was acquired previously. By utilizing a well characterized self-made MUC16 monoclonal antibody, we identified the endogenous interaction between a C-terminal fragment of MUC16 (MUC16C) and β-catenin for the first time, and further elucidated that trans-activation domain of β-catenin is required for this interaction. Such interaction could activate the Wnt/β-catenin signaling pathway by facilitating cytosol-nucleus transportation of β-catenin, consequently induce cell proliferation and the migration, eventually lead to tumorigenesis and metastasis in nude mice. Consistently, knockdown of MUC16 significantly weakened the capabilities of cells for proliferation and migration. Based on our discovery, we suggest that MUC16 appears as an attractive target for the development of effective anticancer drugs.

## INTRODUCTION

Mucin 16 (MUC16) is the largest glycoprotein (3-5 million Da) in the Mucins family, which provides lubrication by forming mucous (chemical) barriers to protect epithelial tissues from foreign insults [1–3]. A common feature of the mucins is variable numbers of tandem repeats with a high proportion of serines and threonines that are modified by *O*-glycosylation [4–6]. However, aberrant expression of mucins can contribute to loss of epithelial cell polarity, and promote epithelial-to-mesenchymal transition (EMT) progression [2], which leads to enhanced cell motility and invasion ability, a key step for tumorigenesis.

MUC16 contains an extracellular N-terminal region, glycosylated tandem repeat domain and a C-terminal portion consisting of a transmembrane region and a 32-residue cytoplasmic tail with several tyrosine, serine and threonine sites for potential phosphorylation [7–9]. Compared with other mucin family members, the expression of MUC16 is very limited [9]. It is expressed by normal bronchial, endometrial, ovarian and corneal epithelial cells [10–13], and has evolved from the proteoglycan, agrin [14]. The extracellular region of over-expressed MUC16 in epithelial ovarian carcinoma can be cleaved from cell surface to become circulating elements in the blood, which is a well-established ovarian cancer marker for clinical diagnosis, known as CA125 [15–20]. Aberrant MUC16 expression can also appear in patients with other types of cancers, including breast, lung and pancreas malignancies [21–23]. During last 30 years, MUC16/CA125 had attracted plenty attention to its clinical application as a cancer biomarker [18, 19]. However, functions and molecular mechanisms of MUC16 are still largely unclear, owing to its relatively late identification and the difficulties in analyzing a protein of its enormous size [8, 9].

Unlike MUC16, the other mucin family member, Mucin 1 (MUC1), has been well studied [2, 3, 6]. Moreover, it was identified to be an oncoprotein which has abilities to promote the molecular process of EMT and lead to increased invasion and metastasis [24–26]. Hallmarks of EMT include the loss of abundance of tight junction proteins or function of E-cadherin as well as concomitant increase in abundance of mesenchymal markers, such as vimentin, fibronectin and N-cadherin [27–31]. According to previous reports, MUC1 can interact with β-catenin, which leads to the translocation of β-catenin into the nucleus [32–34]. The Wnt/β-catenin signaling pathway has been demonstrated to play important roles in the development, promotion of EMT and cancer metastasis [35–37]. Upon this pathway was activated, β-catenin could be stabilized and accumulated in the cytoplasm, eventually translocated into the nucleus to act as a transcriptional coactivator for the TCF/LEF family, therefore to initiate the transcription of Wnt downstream genes required for EMT and metastasis [38–41]. Thus the interaction of MUC1 and β-catenin is important for EMT transformation in cancer cells [42].

Recently, MUC16 has been noticed as a multifunctional molecule with different domains involved in various signaling pathways [43–48]. Some interacting partners of MUC16 have been reported, including Mesothelin and Src family kinases (SFKs) [49–51]. In addition, it was reported that proteolytic cleaved/cell surface MUC16 could interact with Siglec-9 of immune cells, which could act as an anti-adhesive agent to block tumor-immune cell interactions [52]. Interestingly, MUC16 shares some structural similarities with MUC1, it has a SXXXXXSSX motif in its C-terminus similar to MUC1, such a motif is crucial for the interaction between MUC1 and β-catenin [32] (this motif is “**S**AGNGG**SS**L” in MUC1 while that in MUC16 is “**S**VYQPT**SS**S”). Additionally, some studies suggested that MUC16 could play a similar role like MUC1 [34, 53] in promoting tumorigenesis. It was proved to be an important regulator for tumorigenesis and metastasis abilities of epithelial ovarian cancer cells [54–56]. Others suggested that MUC16 could be a key factor to switch E-cadherin to N-cadherin expression at the cell surface [53]. The importance of various C-terminal fragments of MUC16 in cell growth, invasion and/or cell adhesion were reported [44, 57, 58].

In our studies, we applied a self-made mouse monoclonal antibody against MUC16 to investigate whether a short C-terminal fragment (200 amino acids) of MUC16 (MUC16C) containing “**S**VYQPT**SS**S” motif can bind to β-catenin, and the effects of this MUC16C, which was supposed to mimic remaining MUC16 fragment in cells after proteolytic cleavage, on cell proliferation, migration, invasion and metastasis. Our results demonstrated that MUC16/MUC16C could bind to β-catenin at endogenous level, and over-expressed MUC16C could activate the Wnt signaling pathway through stabilization and enhanced cytosol-nucleus translocation of β-catenin. Tumor cells expressing ectopic MUC16C showed the distinct metastatic characteristics.

## RESULTS

### MUC16/MUC16C interacts with β-catenin

**Figure 1:**
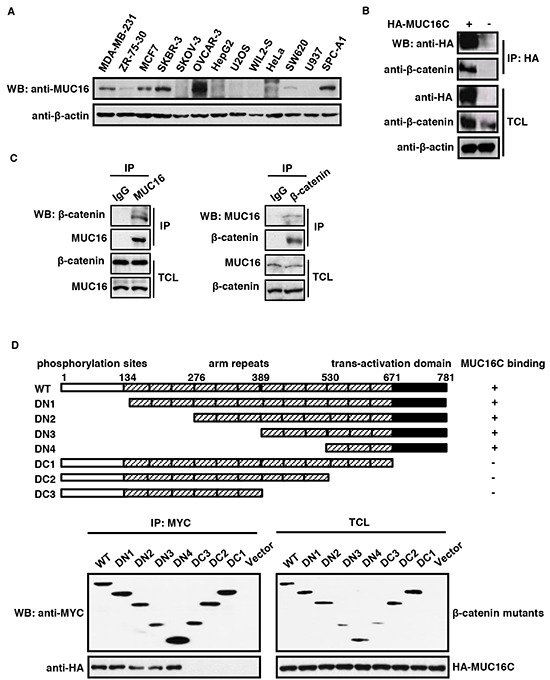
MUC16/MUC16C interacts with β-catenin **A.** Expression profile of MUC16 in different cell lines. The cell lysates were analyzed by Western blot with the self-made mouse anti-MUC16 (40 μg input) or mouse anti-β-actin (8 μg input) antibody respectively. **B.** Ectopically expressed MUC16C interacts with endogenous β-catenin. HeLa cells were transfected with pcDNA3.3-HA-MUC16C or the empty vector as a control. At 24 h post-transfection, cells were lysed and subjected to immunoprecipitation with mouse anti-HA antibody, followed by Western blot with mouse anti-HA and rabbit anti-β-catenin antibodies separately. **C.** MUC16 and β-catenin interact with each other in vivo. Lysates of SKBR-3 cells were subjected to immunoprecipitation with the control IgG, rabbit anti-β-catenin and mouse anti-MUC16 antibodies respectively. The precipitates were then detected with indicated antibodies. **D.** C-terminus of β-catenin is essential for its interaction with MUC16. For the domain mapping experiment, structures of deletion mutants of β-catenin are shown on the top of the panel. Functional domains of β-catenin are indicated above the schema; the remaining fragments of each deletion mutant are shown in the diagram. HEK293T cells were co-transfected with pcDNA3.3-HA-MUC16C and different MYC-β-catenin mutants or the empty vector as a control. At 24 h post-transfection, cells were lysed and subjected to immunoprecipitation with rabbit anti-MYC antibody, followed by Western blot with mouse anti-HA or anti-MYC antibody.

The expression of MUC16 in various cell lines was checked with our self-made MUC16 antibody which can detect specific endogenous MUC16 around 250KD (A full gel picture of Western blot was shown in Supplementary Figure S1). This MUC16 monoclonal antibody was obtain through the hybridoma technology with a C-terminal fragment, and it was well characterized by Western blot (Figure 1A), immunoprecipitation (Figure 1C), shRNA (Figure 5B), immunostaining (Supplementary Figure S2A) etc.. All of these data convinced us that this antibody could recognize endogenous MUC16. It turned out that OVCAR-3 cell line has the highest amount of endogenous MUC16, followed by SKBR-3 cell line, while the other ovarian cancer cell line SKOV-3 has just trace amount of MUC16 (Figure 1A). Next, the interaction between MUC16C or MUC16 and β-catenin was examined by co-immunoprecipitation (Co-IP) assay. We found that endogenous β-catenin was co-immunoprecipitated with ectopically expressed MUC16C in HeLa cells (Figure 1B). Meanwhile in vivo Co-IP showed that endogenous MUC16 and β-catenin in SKBR-3 cells could interact with each other reciprocally (Figure 1C).

Further dissection of MUC16/MUC16C binding site on β-catenin was performed by the domain mapping experiment. We created a series of plasmids expressing wild type β-catenin and its deletion mutants (deletions at N terminus, DN1, DN2, DN3 and DN4; deletions at C terminus, DC1, DC2 and DC3) as shown in the upper panel of Figure 1D. These plasmids were co-transfected with HA-MUC16C into HEK293T cells respectively, followed by Co-IP and Western blot (Figure 1D, bottom panel). HA-MUC16C failed to be precipitated by all three β-catenin mutants deleted with C-terminal transactivation domain, indicating that C-terminus (the trans-activation domain) of β-catenin is required for its interaction with MUC16C (Figure 1D).

### MUC16C promotes β-catenin-dependent transcriptional activity through enhanced cytosol-nucleus transportation

**Figure 2:**
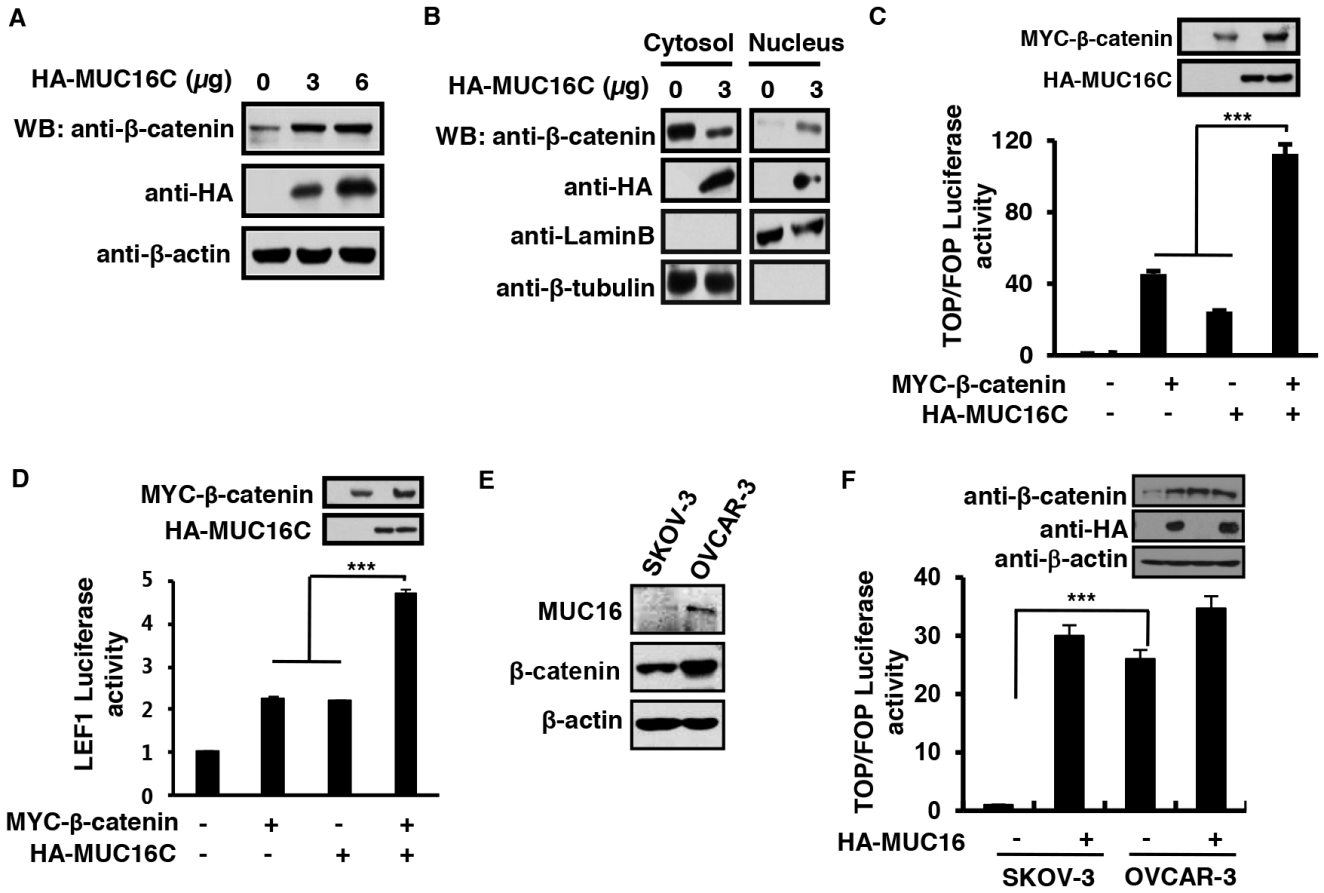
MUC16C promotes β-catenin-dependent transcriptional activity through enhanced cytosol-nucleus transportation **A.** MUC16C increases the protein level of β-catenin. SKOV-3 cells were transfected with different amount of pcDNA3.3-HA-MUC16C vectors (0, 3 and 6 μg). At 24 h post-transfection, total cell lysates were harvested and subjected to Western blot with indicated antibodies. **B.** MUC16C enhances the cytosol-nucleus transportation of β-catenin. SKOV-3 cells were transfected with different amount of pcDNA3.3-HA-MUC16C vectors (0 and 3 μg). At 72 h post-transfection, cells were harvested and subjected to nuclear/cytosol fractionation, followed by Western blot with rabbit anti-β-catenin, mouse anti-HA, goat anti-Lamin B and rabbit anti-β-tubulin antibodies respectively. MUC16C activates β-catenin-dependent TOP/FOP luciferase reporter (C) and LEF1 reporter (D). HEK293T cells were transfected with the expression vectors of MYC-β-catenin and HA-MUC16C alone or in combination, together with pRL-TK and TOP/FOP luciferase reporter **C.** or pGL3-fos-7LEF-luciferase/pCG-LEF1 **D.** At 24 h post-transfection, luciferase activities were determined, and the results are presented as mean±SD of three independent experiments. The protein levels of ectopically expressed β-catenin and MUC16C were analyzed by Western blot. **E.** In ovary cancer cell lines SKOV-3 and OVCAR-3, the protein levels of MUC16 are closely correlated with that of β-catenin. Protein levels were determined by using Western blot. **F.** Wnt signaling is significantly activated in OVCAR-3 cells rather than in SKOV-3 cells. SKOV-3 and OVCAR-3 cells transfected with or without HA-MUC16C, together with TOP/FOP luciferase reporter were determined for relative luciferase activities. ***P<0.0001, student's *T*-test.

The effect of MUC16C on β-catenin was then investigated. We observed that over-expressed MUC16C could elevate endogenous β-catenin expression (Figure 2A). It is well known that β-catenin was accumulated in the nucleus in many cancerous cells to achieve effective transcriptional activity, therefore we examined the role of over-expressed MUC16C on accumulation of β-catenin in the nucleus through the nuclear/cytosol fractionation experiment. Interestingly, we found that over-expressed MUC16C in SKOV-3 cells (Figure 2B) could increase the level of nuclear β-catenin while decrease the level of cytoplasmic β-catenin concomitantly. Meantime, over-expressed MUC16C localized in the nucleus was observed by immunostainning (Supplementary Figure S2B). Furthermore, over-expression of MUC16C and β-catenin could additively promote β-catenin-dependent transcriptional activity with either TOP/FOP luciferase reporter (Figure 2C) or LEF1 luciferase reporter (Figure 2D) assays. As MUC16 is expressed quite differently in ovary cancer cell lines SKOV-3 and OVCAR-3 (Figure 1A), we detected β-catenin in these two cell lines and found that the protein levels of MUC16 were closely correlated with that of β-catenin (Figure 2E). Consistently, TOP/FOP luciferase reporter was significantly activated in OVCAR-3 cells with high level of MUC16 rather than in SKOV-3 cells with low level of MUC16, however, rescuing expression of HA-MUC16C restored the ability of SKOV-3 cells to activate TOP/FOP luciferase reporter (Figure 2F). Over all, MUC16C could promote β-catenin-dependent transcriptional activity through enhanced cytosol-nucleus transportation of β-catenin.

### MUC16C up-regulates expression of Wnt target genes and promotes cell proliferation

**Figure 3:**
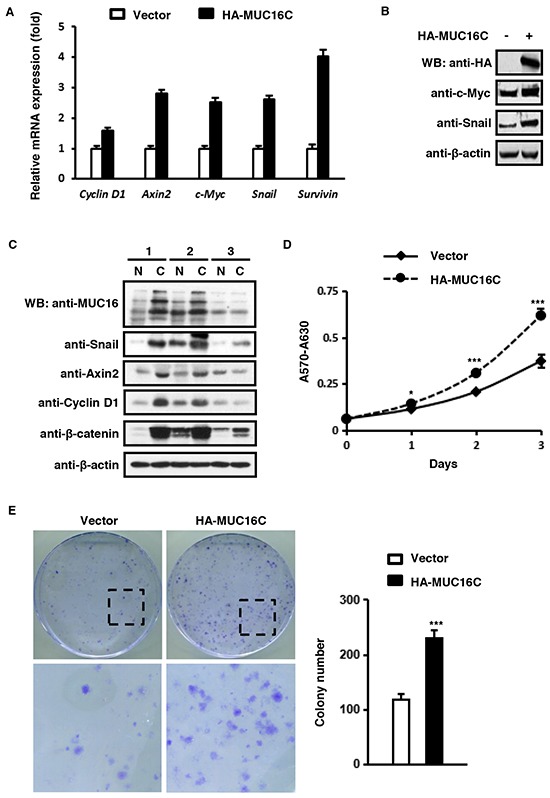
MUC16C up-regulates expression of Wnt target genes and promotes cell proliferation **A.** MUC16C increases mRNA levels of classic downstream genes of the Wnt signaling pathway. SKOV-3 cells were infected with lentivirus containing pBOBI-HA-MUC16C or pBOBI vector as a control. At 36 h post-infection, cells were harvested and the mRNA levels of *Cyclin D1*, *Axin2*, *c-Myc*, *Snail* and *Survivin* were determined by quantitative RT-PCR. **B.** MUC16C up-regulates protein levels of Wnt target genes c-Myc and Snail. SKOV-3 cells were processed as in (A). At 36 h post-infection, cells were lysed and subjected to Western blot with indicated antibodies. **C.** The expression of Wnt target genes is relative to MUC16 in three human breast cancer samples. The homogenates of these three pathological samples (N=normal, C=carcinoma) were analyzed by Western blot with indicated antibodies. **D.** MUC16C promotes cell proliferation as assessed by MTT assay. SKOV-3 cells were processed as in (A). Proliferation of cells was measured by MTT staining during a three-day culture period. Results were shown as mean±SEM of three independent experiments. Significance was calculated by the student's *T*-test (*P<0.01, ***P<0.0001). **E.** MUC16C promotes cell proliferation as assessed by colony formation assay, in which 2 × 10^2^ cells of SKOV-3 stably expressing HA-MUC16C or the empty vector as a control were seeded on 100 mm culture dishes. After 2 weeks, colonies were fixed and stained with crystal violet. Colonies numbers were counted and showed as mean±SD of three independent experiments. Significance was calculated by the student's *T*-test (***P<0.0001).

Based on the facts that β-catenin is the key mediator of the Wnt signaling pathway, and MUC16 could activate β-catenin transcriptional activity, we decided to investigate the effect of ectopic expression of MUC16C on the Wnt downstream genes. Results from fluorescence quantitative RT-PCR showed that the mRNA levels of five Wnt down-stream genes, *Cyclin D1*, *Axin2*, *c-Myc*, *Snail* and *Survivin*, were up-regulated in SKOV-3 cells with over-expressed MUC16C (Figure 3A). In addition, results of Western blot indicated that expression of both c-Myc and Snail was increased when MUC16C was over-expressed in SKOV-3 cells (Figure 3B). Furthermore, we checked the protein level of MUC16, β-catenin and three Wnt downstream genes, Snail, Axin2 and Cyclin D1 in three sets of human breast cancer samples by Western blot (Figure 3C, Supplementary Table S1). It turned out that, the protein levels of β-catenin, Snail, Axin 2 and Cyclin D1 in the carcinoma tissue (C) were elevated along with MUC16 in the number 1 and number 2 breast cancer samples, compared with the counterparts of normal tissue (N). While in the number 3 sample, MUC16 did not obviously increase in the carcinoma tissue, and those proteins showed no changes coincidentally (Figure 3C).

To understand the effect of elevated Wnt downstream genes caused by over-expressed MUC16C on cellular functions, we performed MTT and colony formation assays to evaluate cell proliferation. Results of MTT assay indicated that MUC16C over-expression could remarkably promote cell proliferation (Figure 3D). Consistently, the number of colonies was significantly higher with SKOV-3 cells expressing MUC16C when compared to that of the control cells (Figure 3E). Collectively, these results indicated that ectopic expression of MUC16C can upregulate Wnt target genes and promote cell proliferation.

### MUC16C promotes cell migration and invasion

**Figure 4:**
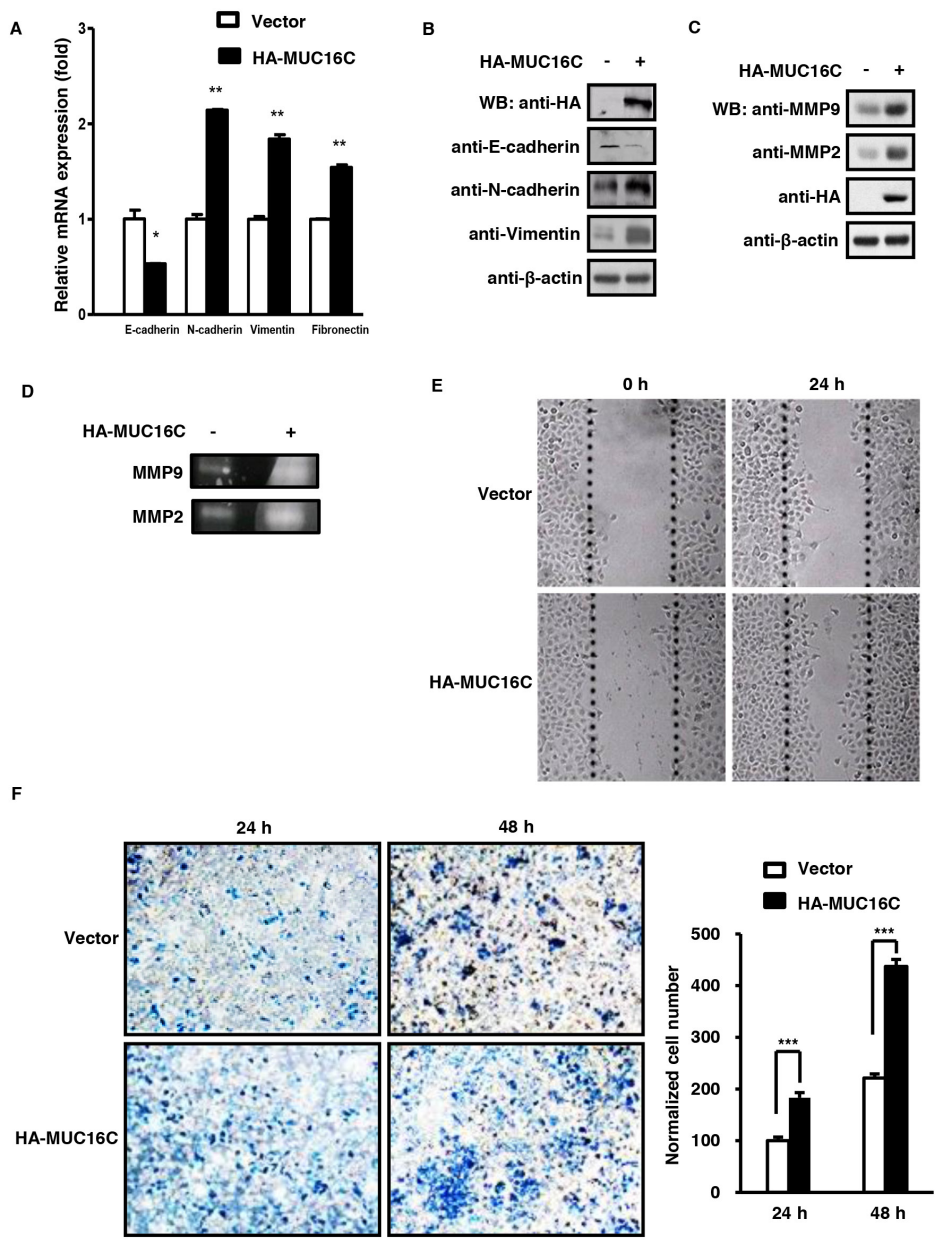
MUC16C promotes cell migration and invasion **A.** MUC16C influences mRNA levels of novel EMT markers like E-cadherin, N-cadherin, Vimentin and Fibronectin. SKOV-3 cells with or without over-expressed MUC16C were harvested at 36 h post-infection and the mRNA levels of above EMT markers were determined by RT-PCR. **B.** MUC16C influences protein levels of EMT markers. SKOV-3 cells described in (A) were lysed at 36 h post-infection, followed by Western blot with indicated antibodies. MUC16C promotes expression of MMP family members, MMP2 and MMP9, as assessed by Western blot in **C.** or gelatin zymography assay in **D.**. SKOV-3 cells for assays in (D) were cultured further in the serum-free medium for another 48 h, and then the culture medium was collected for detecting activated MMP9 and MMP2. **E.** MUC16C promotes migration of SKOV-3 cells. The SKOV-3 cells described above in (A) were subjected to wound healing assay. Photographs were taken at different time points (0, 24 h). **F.** MUC16C promotes invasion of SKOV-3 cells. The SKOV-3 cells described above in (A) were subjected to transwell invasion assays. Data are reported as normalized number of cells that invaded through the transwell membrane relative to that of the control (100%), and represent the mean mean±SD of three independent experiments. Significance was calculated by the student's *T*-test (***P<0.0001).

Next, we checked the levels of mRNA and protein for several EMT markers to find out if MUC16C plays a role in tumor cell migration and invasion. Quantitative RT-PCR indicated that mRNA level of *N-Cadherin, Vimentin* and *Fibronectin* was increased while *E-cadherin* decreased in SKOV-3 cells with over-expressed MUC16C (Figure 4A). Accordingly, the protein levels of both N-cadherin and Vimentin were increased while that of E-cadherin was decreased when MUC16C was over-expressed in SKOV-3 cells (Figure 4B). In addition, both Western blot (Figure 4C) and gelatin zymography assays (Figure 4D) showed that the protein levels of MMP9 and MMP2, important markers for cell migration and metastasis, were also increased in SKOV-3 cells with over-expressed MUC16C. Furthermore, in comparison to the control cells, over-expressed MUC16C conferred SKOV-3 cells enhanced migration ability in the wound healing assay (Figure 4E). In addition, over-expressed MUC16C strengthened invasion of SKOV-3 cells as indicated in the transwell invasion assay (Figure 4F). Taken together, these results suggest that ectopic expression of MUC16C can strongly enhance migration and invasion properties of SKOV-3 cells. There is also a close correlation between MUC16/MUC16C overexpression and EMT phenotypes (Supplementary Figure S3).

### *MUC16* knockdown reduces proliferation and migration of SKBR-3 cells

**Figure 5:**
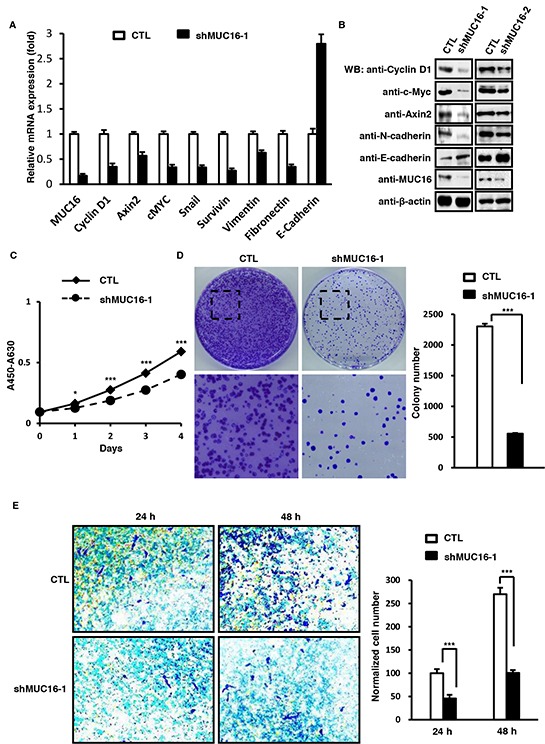
MUC16 knockdown reduces proliferation and metastasis of SKBR-3 cells **A.** *MUC16* knockdown influences mRNA levels of classic Wnt target genes and EMT markers. SKBR-3 cells were infected with lentivirus containing pLV-LacZ (CTL) or pLV-MUC16 (shMUC16-1). At 36 h post-infection, cells were harvested and the mRNA levels of *MUC16*, *Cyclin D1*, *Axin2*, *c-Myc*, *Snail*, *Survivin*, *Vimentin*, *E-cadherin* and *Fibronectin* were determined by RT-PCR. **B.** *MUC16* knockdown in SKBR-3 cells decreases protein levels of Wnt target genes. SKBR-3 cells were transfected with two independent shRNA against MUC16 (shMUC16-1 and shMUC16-2) individually. At 36 h post-infection, cells were lysed and subjected to Western blot with indicated antibodies. **C.** *MUC16* knockdown in SKBR-3 cells inhibits cell proliferation as assessed by CCK8 test. Proliferations of cells described above were measured by CCK8 staining during a four-day culture period. The results were shown as mean±SEM of three independent experiments. Significance was calculated by the student's *T*-test (*P<0.01, ***P<0.0001). **D.** *MUC16* knockdown in SKBR-3 cells inhibits cell proliferation as showed by colony formation assay. 2 × 10^2^ of SKBR-3 cells were seeded on 100 mm culture dishes. After 2 weeks, colonies were fixed and stained with crystal violet. Numbers of colonies were counted and showed as mean±SD of three independent experiments. Significance was calculated by the student's *T*-test (***P<0.0001). **E.** *MUC16* knockdown inhibits invasion of SKBR-3 cells. The SKBR-3 cells described above in (A) were subjected to transwell invasion assays. Data are reported as normalized cell number that invaded through the transwell membrane relative to the empty vector control (100%), and represented as mean±SD of three independent experiments. Significance was calculated by the student's *T*-test (***P<0.0001).

In order to further understand the function of MUC16/MUC16C, we constructed the pLV lentiviral system to knock down *MUC16* in SKBR-3 cells. Along with decrease of MUC16 mRNA, the mRNA levels of the Wnt downstream genes (*Cyclin D1*, *Axin2*, *c-Myc*, *Snail* and *Survivin*) and two EMT markers (*Vimentin* and *Fibronectin*) were also decreased, while the other EMT marker, *E-cadherin* was increased (Figure 5A). Also, the protein levels of three Wnt downstream genes, Cyclin D1, c-Myc and Axin2, were decreased in *MUC16* knockdown SKBR-3 cells as confirmed by two independent shRNA against *MUC16* (Figure 5B). The CCK8 test indicated that knocking down MUC16 would reduce the proliferation of SKBR-3 cells (Figure 5C). This was supported by the results of colony formation assay, in which the *MUC16* knockdown SKBR-3 cells showed weakened ability for colony formation in comparison with that of the control cells (Figure 5D). Additionally, knockdown of *MUC16* gene in SKBR-3 cells would inhibit its migration and invasion ability (Figure 5E). Thus we concluded that *MUC16* knockdown could reduce cell proliferation and migration.

### Over-expressed MUC16C enhances tumorigenesis and metastasis capability of SKOV-3 cells in vivo

**Figure 6:**
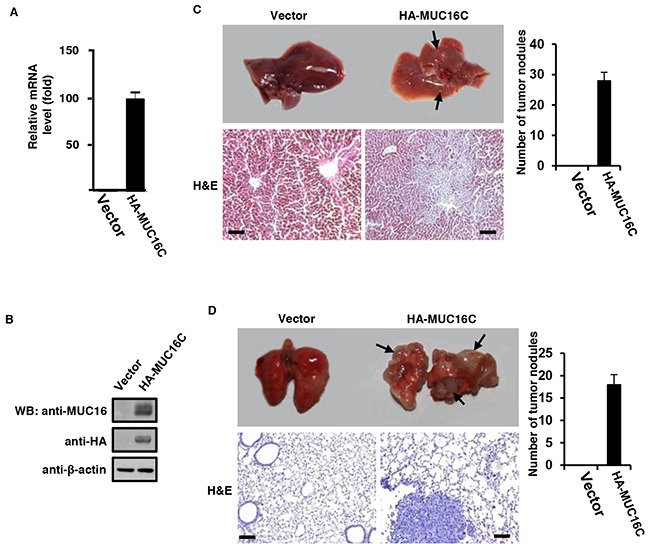
Over-expressed MUC16C enhances metastasis capability of SKOV-3 cells in vivo 1 × 10^6^ of SKOV-3 cells with or without over-expressed MUC16C were injected intravenously into nude mice (*n*=5) once a week for consecutive 3 weeks. After another 5 weeks, mice were sacrificed to analyze their main organs and tissues. **A.** Livers from nude mice described above were homogenated followed by Western blot with antibodies indicated. **B.** Livers from nude mice described above were homogenated and then the total RNA was extracted for fluorescence quantitative RT-PCR analysis of the RNA level of *MUC16*/*MUC16C*. Representative photographs (upper) of livers **C.** and lungs **D.** from nude mice described above and the corresponding histological analyses (lower) are shown. Sections were stained with H&E. The black arrows indicate the metastasis stoves. *Scale bars* 100 μm. Metastatic tumor nodules on the surfaces of livers and lungs were counted and statistical results were presented as histograms in the right (*n*=5).

To investigate the effect of over-expressed MUC16C on metastasis in vivo, we carried out the metastatic tumor formation experiment with nude mice. First, SKOV-3 cells were stably expressed with MUC16C by using pBOBI lentivirus system. Then single cell suspension (1×10^6^) was injected intravenously into nude mice. After 35 days, mice were sacrificed and analyzed. In the mice liver of the experimental group, over-expressed MUC16C (HA-MUC16C) was confirmed by RT-PCR assays (Figure 6A) and Western blot (Figure 6B). Results indicated that both RNA and protein levels of MUC16C increased rigorously in the mice of experimental group (HA-MUC16C) in comparison with that of the control group (vector).

On the other hand, visible tumor metastasis stoves were observed on the surface of livers and lungs of over-expressed MUC16C group (Figure 6C and 6D) while that of the control group looked normal. Further analysis with hematoxylin and eosin (H&E) staining of the metastasized livers and lungs also revealed the enhanced metastasis capability of SKOV-3 cells stably expressing MUC16C (Figure 6C and 6D). Taken together, these results suggest that over-expressed MUC16C can induce remarkable metastasis in vivo.

## DISCUSSION

Aberrant over-expression of mucins is associated with diverse human carcinomas [59]. CA125, a well known clinical tumor marker used to monitor epithelial ovarian cancer, was the cleavage fragment arising from the extracellular domain of up-regulated MUC16. Due to obstacles to work with its large mass, limited knowledge about the mechanisms and functions of MUC16 was acquired previously. Some groups relied on the C-terminus of MUC16 to understand its function in signaling pathways [44, 57, 58]. We took similar approaches by using a C-terminal fragment (200 amino acids) of Mucin 16 (MUC16C), and attempted to reveal possible functions played by presumed remaining MUC16 fragment after proteolytic cleavage at its extracellular domains. According to previous studies, MUC16 can promote tumorigenesis and metastasis while inhibit anti-cancer immune responses.

To support our research, we made great effort to produce a mouse anti-MUC16 monoclonal antibody. Results of immunoprecipitation, Western blot, knockdown assays, immunostaining (Figure 1A, 1C, 5B, 6B and S2) and beyond confirmed that our self-made MUC16 antibody could effectively recognize endogenous MUC16. By applying this MUC16 antibody, we performed Co-IP assays with β-catenin antibody reciprocally to show endogenous interaction between MUC16 and β-catenin for the first time. We further performed the domain mapping experiment, and found that C-terminus (the trans-activation domain) of β-catenin is required for its interaction with MUC16.

The meaning of this interaction was then investigated. As shown in Figure 2A and 2B, over-expressed MUC16C could elevate the protein level of β-catenin and enhance its translocation into nucleus. That translocation of β-catenin into nucleus is the central event to activate the canonical Wnt/β-catenin dependent signaling pathway, therefore to transactivate the Wnt downstream genes. Spontaneously, we wanted to see if enhanced translocation of β-catenin from the cytoplasm into the nucleus caused by over-expressed MUC16C can activate the Wnt downstream genes. We discovered that ectopically expressed MUC16C could up-regulate expression of the Wnt target genes such as Axin2, Cyclin D1, c-Myc, Snail and Survivin as illustrated in Figure 3A and 3B. In addition, MUC16C exhibited the ability to promote tumorigenesis by increasing cell proliferation, migration and metastasis (Figure 3, 4, 5 and 6).

The capability of MUC16C in promoting tumorigenesis especially metastasis was confirmed in the animal model. In nude mice injected intravenously with SKOV-3 cells stably expressing MUC16C, we observed visible tumor metastasis stoves in livers and lungs. The H&E staining confirmed the presence of tumors in those tissues. Different from injecting mice subcutaneously and intraperitoneal with SKOV-3 cells expressing the C-terminus fragment of MUC16 by previous studies [56], our study might mimic the EMT processes in vivo through mice tail vein intravenously injection.

Another interesting observation was made by our studies that ectopically expressed MUC16C can be partially localized at the nucleus (Figure 2B and Supplementary Figure S2B), it brought up the possibility that MUC16C could enter the nucleus along with β-catenin. In order to confirm this possible role of MUC16C in the cytosol-nucleus translocation of β-catenin, more subtle and cautious experiments would be required in future.

In summary, our studies demonstrated that MUC16 is capable of promoting tumorigenesis as indicated by previous studies, and MUC16 knockdown weakens relevant tumorigenetic properties. Based on our results, we proposed that this effect of MUC16 might be achieved by facilitating the accumulation of β-catenin in the nucleus for transactivation of the Wnt downstream genes required by EMT process. This discovery further convinces us that MUC16 could be an attractive therapeutic target besides its clinical diagnostic application. In fact, the efforts with the novel antibody approach targeting at MUC16 is under way, including our effort.

## MATERIALS AND METHODS

### Plasmids and antibodies

The pcDNA3.3-HA-MUC16C and pBOBI-HA-MUC16C expression vectors were constructed by inserting the C-terminal fragment (42925-43524bp) of human MUC16 into respective vectors. To knockdown MUC16, lentiviral vector pLV-based two independent shRNAs against MUC16 were constructed. The targeting sequences for shMUC16-1 and shMUC16-2 are 5′-GGAGCAAGTCTTTCTAGATAA-3′ and 5′-CAGATAAGACCTTGGCCAG -3, individually. pLV-LacZ was used as a control.

Rabbit anti-β-catenin, mouse anti-HA, mouse anti-E-Cadherin, mouse anti-N-Cadherin, mouse anti-Snail, mouse anti-c-Myc monoclonal antibodies were purchased from Cell Signaling Technology (Danvers, MA). Mouse anti-β-actin antibody was purchased from Santa Cruz Biotechnology, Inc. Goat-anti-mouse and goat anti-rabbit immunoglobulin G (IgG) horseradish peroxidase conjugates were purchased from Pierce Biotechnology, Inc.

### Cell culture

The human ovarian cancer cell SKOV-3 and breast cancer cell line SKBR-3 were maintained in McCoy's 5a (GIBCO) medium supplemented with 10% fetal bovine serum (FBS), 100 IU penicillin, 100 mg/ml streptomycin at 37°C in a humidified incubator containing 5% CO2. OVCAR-3 was cultured in the same conditions except that the medium was RPMI-1640 (GIBCO) supplemented with 20% FBS. SPC-A1 was maintained in RPMI-1640 supplemented with 10% FBS while other cell lines were cultured in respective conditions recommended by ATCC.

### Transient transfection and lentivirus packaging

Cell transient transfection was performed by using Lipofectamine 2000 (Invitrogen) according to the manufacturer's instructions.

To produce lentiviruses, HEK293T cells were transfected with pLV or pBOBI lentiviral vector together with packaging vectors pVSV-G, pRSV-Rev and pMDL gag/pol RRE. At 30 h post-transfection, the virus-containing supernatant medium was collected and used for further infection.

### Immunoprecipitation and western blot analysis

Cell lysis and immunoprecipitation were carried out as previously described [60]. Briefly, cells were lysed with lysis buffer (20 mM Tris-HCl [pH 7.4], 150 mM NaCl, 1% Triton X-100, 1 mM phenylmethanesulfonyl fluoride, 10 mg/ml leupeptin, 2 mg/ml aprotinin, 10 mM NaF, 1 mM Na_3_VO_4_). The protein concentration was determined by using bicinchoninic acid (BCA) assay. For immunoprecipitation, cell lysates were incubated with respective antibodies overnight at 4°C, then protein A/G beads were added into the lysates and incubated for another 3 h. Levels of proteins in total cell lysate and precipitants were analyzed on the separate gels.

### Colony formation assay

For colony formation assay, cells were seeded on 100 mm dishes. After 2 weeks, cells were fixed, viable colonies were analyzed after staining with 0.2% crystal violet, and colony formation was monitored under an inverted phase contrast microscope (Nikon TE2000). Numbers of colony formation were counted and showed as mean±SD.

### Fluorescence quantitative Real-Time PCR

Total RNA was extracted using TRIzol reagent (Invitrogen, Carlsbad, CA) according to the manufacturer's instructions. First strand cDNA was synthesized from the total RNA using an oligo (dT) primer and Moloney murine leukemia virus reverse transcriptase (TaKaRa, Shiga, Japan). Real-time PCR was performed on the Rotor-Gene 6000 (Corbett Research, Mortlake, Australia). Each sample was run in triplicate. Data analysis was performed with the Rotor-Gene 6000 series software 1.7 (Corbett Research, Mortlake, Australia).

**Table:** The relative RNA amounts were normalized to β-actin mRNA. The primer sequences were seen as below

Gene	Forward (5′-3′)	Reverse (5′-3′)
MUC16	AGCACCCAGCACTTCTACCT	GCGCATCCTCAATATTCCTT
Vimentin	GAGAACTTTGCCGTTGAAGC	GCTTCCTGTAGGTGGCAATC
E-Cadherin	CCCATCAGCTGCCCAGAAAATGAA	CTGTCACCTTCAGCCATCCTGTTT
N-cadherin	CCTTTCAAACACAGCCACGG	TGTTTGGGTCGGTCTGGATG
Fibronctin	CAGTGGGAGACCTCGAGAAG	TCCCTCGGAACATCAGAAAC
Cyclin D1	CCGTCCATGCGGAAGATC	GAAGACCTCCTCCTCGCACT
Survivin	ACCACCGCATCTCTAC	TCCTCTATGGGGTCGT
Axin2	GCAAACTTTCGCCAACCGTG	CTCTGGAGCTGTTTCTTACTGCCC
c-Myc	CCCTTGCCGCATCCACG	CGAGGTCATAGTTCCTGTTGGTG
Snail	GACCCCAATCGGAAGCCTAACTA	AGCCTTTCCCACTGTCCTCATCT
β-actin	TCCCTGGAGAAGAGCTACG	GTAGTTTCGTGGATGCCACA

### Gelatin zymography

Activation of MMP-2 and MMP-9 was assessed by gelatin zymography. Cells were cultured in the serum-free medium for 48 h, then the culture supernatants were concentrated and run on the 8% polyacrylamide gel containing 1% gelatin by electrophoresis. Next, the gel was incubated with 2.5% Triton X-100 for 30 min to remove SDS, before the gelatinase activity on the gel was initiated by incubating with development solution (50 mM Tris–HCl [pH7.8], 200 mM NaCl, 5 mM CaCl2, 0.02% Brij-35) for 18 h at 37°C, gels were then stained with Coomassie Blue.

### Transwell invasion assay

Cells were seeded on the trans-well inserts (poly carbonate filter with 8 μm pores, Millipore, Cat No. PIEP 12R48) filled with serum-free medium. The bottom chamber contained 500 μl medium supplemented with 10% FBS as chemo attractant. After 24 h or 48 h culture, cells that remained inside the filter were removed completely with a cotton swap. Cells that migrated to the lower surface of the filter membrane were fixed with 0.4% methanol, and stained with 0.4% trypan blue. Filters were air dried, and photos were taken with an inverted phase contrast microscope (Nikon TE2000).

### Nuclear/cytosol fractionation

Nuclear and cytoplasmic lysates were prepared using a nuclear/cytosol Fractionation Kit (Bio Vision, CA, US). Cells were collected and re-suspended with 0.2 ml CEB-A mix containing DTT and protease inhibitors. After incubation with 11 μl CEB-B for 1 min, cell lysates were then centrifuged, and the supernatant of the cytoplasmic extract was collected. Next, the remaining cell pellet was re-suspended with 100 μl NEB mix, followed by centrifugation, and then the supernatant of the nuclear extract was collected.

### Luciferase assay

The luciferase reporter assay was carried out using the Dual-Luciferase Reporter Assay System (Promega, Madison, WI, US). Cells were seeded on the 12-well plates. Cells were transfected with respective expression vectors, and co-transfected with either TOP flash (3× TCF4 binding sites)/FOP flash (3× mutated TCF4 binding sites) expression vectors, or with LEF1/LEF1 reporter. pRL-TK (Renilla-TK-luciferase vector, Promega) was co-transfected as the intrinsic control. TCF-mediated transcriptional activity was determined by the ratio of TOP flash/FOP flash luciferase activity, each was normalized to the luciferase activities of the pRL-TK reporter. The experiments were performed in triplicate.

### MTT assay and CCK8 assay

Cells (2000/well) were seeded into 96-well plates and cultured for desired time frame, then stained with 100 μl of 0.5 mg/ml sterile3-(4,5-dimethythiazol-2-yl)-2,5-diphenyltetrazoliumbromide (MTT, Sigma, St. Louis, MO, US) at indicated time points for 4 h at 37°C, followed by removal of the culture medium and addition of 150 μl of dimethysulfoxide (Sigma). The absorbance was measured at 570 nm and 630 nm separately. All tests were performed in triplicate. The CCK8 assay is similar, while cells were stained with 10 μl of CCK8 solution (Dojindo Molecular Technologies Inc., China) for 2 h at 37°C, then the absorbance was measured at 450 nm and 630 nm separately.

### Wound healing assay

Cells were seeded into 35 mm culture plate in a density that could reach 70-80% confluence as a monolayer after 24h incubation. Without changing the medium, culture cells were scratched gently and slowly across the center of the well with a new 10 μl pipette tip. After scratching, the plate was gently washed twice with medium to remove detached cells. Remaining cells were replenished with fresh medium, and continually cultured for additional 24 h (or as the time required), then cells were washed twice with 1x PBS for observation under an inverted phase contrast microscope (Nikon TE2000). Set the same configurations of the microscope when taking pictures for different views of cells.

### Nude mice metastatic tumor formation assay

4-5-week-old female athymic BALB/c nude mice were housed under specific pathogen free (SPF) conditions in compliance with the Institutional Animal Care and Use Committee at Xiamen University. Single cell suspension (1 × 10^6^) of SKOV-3 cells, infected with lentivirus expressing HA-MUC16C or control lentivirus, was injected intravenously into nude mice once a week for consecutive 3 weeks. Each group contained 5 mice. After another 5 weeks, mice were sacrificed and examined. Metastasized mouse liver and lung were embedded in OCT (Sakura Finetek Inc, Torrance, CA 90501 US) for frozen section procedure, the sections were then stained with hematoxylin and eosin (H&E).

### Statistical analysis

Values were expressed as mean±SD or mean±SEM. To determine significant differences in the data, the two-tailed unpaired Student's T test was applied. Differences were considered to be statistically significant at P<0.05.

## SUPPLEMENTARY FIGURES AND TABLE



## Abbreviations

EMT: epithelial mesenchymal transition
SFKs: Src family kinases
co-IP: co-immuniprecipitation
TCL: total cell lysate
WB: Western blot
