# Supplementary material for: C-terminus of MUC16 activates Wnt signaling pathway through its interaction with β-catenin to promote tumorigenesis and metastasis
Source: Oncotarget. 2016 May 5;7(24):36800–13. doi: 10.18632/oncotarget.9191 (PMC5095040; doi:10.18632/oncotarget.9191)
Supplement: Supplementary file 1 [file oncotarget-07-36800-s001.pdf]

SUPPLEMENTARY FIGURES AND TABLE

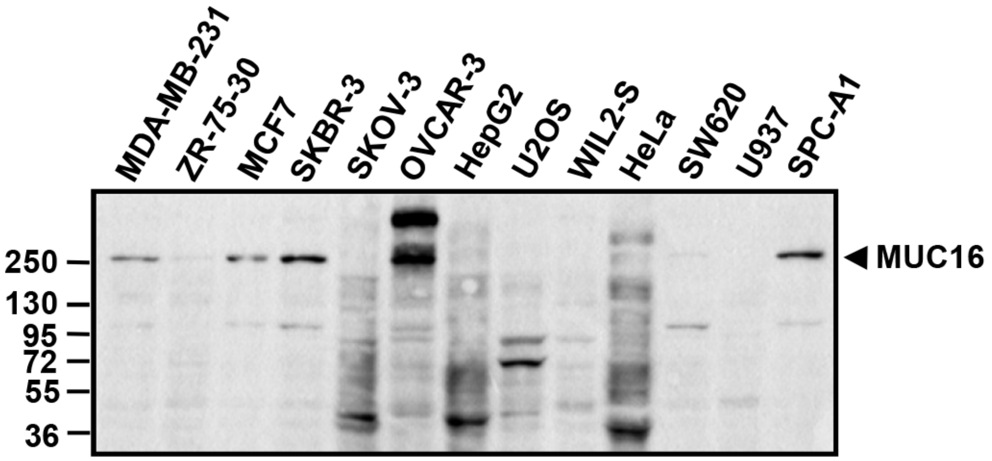

Supplementary Figure S1: A full gel picture of Western blot indicated the molecular weights of endogenous MUC16 in different cell lines.

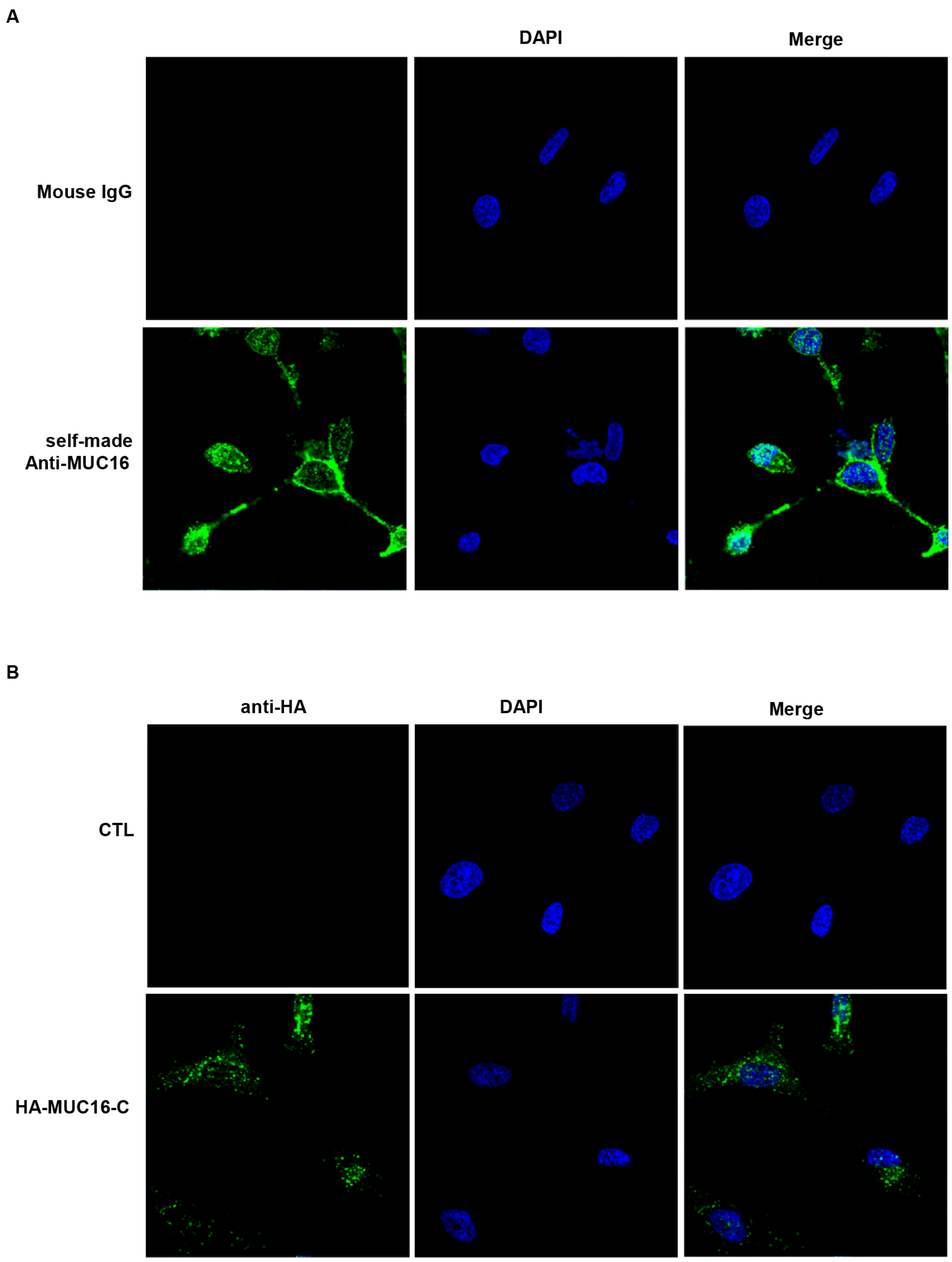

Supplementary Figure S2: Cellular localization of MUC16/MUC16C was indicated by immunofluorescence staining. **A.** Endogenous MUC16 was detected by our anti-MUC16 antibody. **B.** Over-expressed MUC16C was partially localized into the nucleus. SKOV-3 cells were transfected with pcDNA3.3-HA-MUC16C (HA-MUC16C) or the empty vector as a control (CTL). HA-MUC16C proteins were stained with mouse anti-HA primary antibody followed by Alexa Fluor 488 conjugated donkey anti-mouse secondary antibody (green) while the nuclei were indicated by DAPI staining (blue). Images were acquired with a confocal laser-scanning microscope (Zeiss, LSM780).

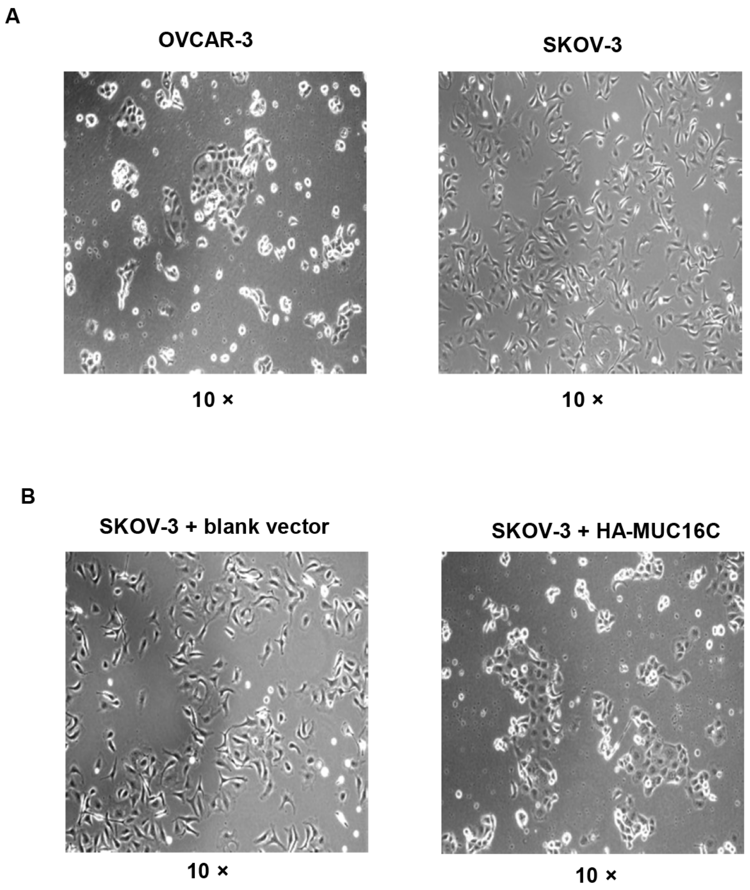

Supplementary Figure S3: There is a close correlation between MUC16 expression and EMT phenotypes. A. Comparison of the morphologies between OVCAR-3 cells with MUC16 highly expressed and SKOV-3 cells with MUC16 lowly expressed; SKOV-3 cells showed better attachment and adhesion ability versus OVCAR-3 cells. **B.** The cell morphology of SKOV-3 cells transfected with the control vector and MUC16C expression vector. SKOV-3 cells transfected with MUC16C showed less attachment and adhesion ability.

Supplementary Table S1: The information about the three breast cancer samples

NO.	Gender	Age	Pathology disgnosis	Grade*	TNM**	Type
1	F	52	Invasive ductal carcinoma	2	T2N0M0	Malignant
2	F	48	Invasive ductal carcinoma	2	T2N0M0	Malignant
3	F	38	Invasive ductal carcinoma	2	T2N0M0	Malignant

* The grade 2 in pathology disgnosis is equivalent to moderately-differentiated under microscope
** TNM grading:
T2 - Tumor invades muscularis propria
N0 - No regional lymph node metastasis
M0 - No distant metastasis
